# *MUMOTT*: a Python package for the analysis of multi-modal tensor tomography data

**DOI:** 10.1107/S1600576725007289

**Published:** 2025-09-12

**Authors:** Leonard C. Nielsen, Mads Carlsen, Sici Wang, Arthur Baroni, Torne Tänzer, Marianne Liebi, Paul Erhart

**Affiliations:** a Department of Physics Chalmers University of Technology Gothenburg Sweden; b Photon Science Division Paul Scherrer Institute (PSI) Villigen Switzerland; c Institute of Materials, École Polytechnique Fédérale de Lausanne, Lausanne, Switzerland; Argonne National Laboratory, USA

**Keywords:** small-angle X-ray scattering, wide-angle X-ray scattering, tensor tomography, reconstruction, software

## Abstract

Small- and wide-angle X-ray scattering tensor tomography are powerful methods for studying anisotropic nanostructures in a volume-resolved manner and are becoming increasingly available to users of synchrotron facilities. The analysis of such experiments requires advanced procedures and algorithms, which creates a barrier for the wider adoption of these techniques. Here, in response to this challenge, we introduce the *MUMOTT* package. It is written in Python, with computationally demanding tasks handled via just-in-time compilation using both CPU and GPU resources. The package has been developed with a focus on usability and extensibility, while achieving a high computational efficiency. Following a short introduction to the common workflow, we review key features, outline the underlying object-oriented framework and demonstrate the computational performance. By developing the *MUMOTT* package and making it generally available, we hope to lower the threshold for the adoption of tensor tomography and to make these techniques accessible to a larger research community.

## Abstract 2

The *MUMOTT* Python package facilitates the analysis of small- and wide-angle X-ray scattering tensor tomography data, using CPU and GPU acceleration to simplify complex computational tasks. Designed for ease of use, extensibility and efficiency, *MUMOTT* aims to lower barriers to adopting tensor tomography methods within the wider research community.

## 1. Introduction

The properties of numerous materials depend on the hierarchical organization of their basic building blocks, ranging from the nanometre to the micrometre scale. Examples include plant materials assembled from cellulose and lignin (Fratzl & Weinkamer, 2007 ▸), bone constructed of assemblies of mineralized collagen fibrils (Reznikov *et al.*, 2014 ▸), and polymeric materials, such as semi-crystalline polymers (Schrau­wen *et al.*, 2004 ▸; Stribeck *et al.*, 2008 ▸; Tang *et al.*, 2007 ▸) and liquid-crystalline polymers composed of rigid macromolecules (Gantenbein *et al.*, 2018 ▸). The study of structure–property relationships of hierarchical materials for applications in biology, the biomedical field or polymer engineering therefore relies on accurate structural characterization from a wide range of techniques. Here, X-ray techniques are of particular interest as they can provide volume-resolved nanostructural information in macroscopic samples thanks to their high penetration depth and non-destructive nature. Methods such as X-ray absorption and phase contrast computed tomography (CT) therefore play an important role in providing high-resolution densimetric measurements of 3D samples (Endrizzi, 2018 ▸; Ou *et al.*, 2021 ▸).

In addition to the densimetric fields, the arrangement of nanostructural elements, in particular their direction and degree of alignment, is important for many mechanical and functional properties on larger length scales. This situation introduces a further methodological challenge: bridging the length scales between nanostructural building blocks and the macroscopic specimen. One approach to this challenge involves probing nanostructure orientation in a volume-averaged manner using techniques based on polarization, scattering, diffraction or magnetic relaxation (Georgiadis *et al.*, 2016 ▸). For spatially resolved information, X-ray and neutron diffraction approaches can be used, including directional dark-field (DDF) imaging (Jensen *et al.*, 2010 ▸; Busi *et al.*, 2023 ▸), which probes the orientation on the micrometre scale through the integrated scattering signal, as well as scanning small- and wide-angle scattering techniques, which probe the nanoscale structures. Specifically, small-angle X-ray scattering (SAXS) probes the spatial variation of the electron density, providing information on structural elements with characteristic length scales in the range of tens to hundreds of nanometres. It thus provides access to information regarding the structural organization and orientation of the material at the corresponding length scales, while X-ray diffraction (XRD) (in this paper called wide-angle X-ray scattering, WAXS) probes atomic distances and crystal structure. Whereas DDF imaging comprises a family of full-field imaging methods, SAXS and WAXS can be used as scanning imaging techniques in which the sample is raster-scanned with a focused X-ray beam, providing an image of the sample consisting of a 2D diffraction pattern in each pixel. Tomographic reconstruction of such measurements using isotropically scattering samples is known as XRD-CT and is frequently used in both the SAXS (Stribeck *et al.*, 2006 ▸; Schroer *et al.*, 2006 ▸) and WAXS (Kleuker *et al.*, 1998 ▸; Stock *et al.*, 2008 ▸; Bleuet *et al.*, 2008 ▸) regimes at synchrotron X-ray sources.

To access the orientation information of the underlying ultrastructure within a 3D specimen, tomographic methods can be extended from the reconstruction of scalar fields to tensor fields describing the directionality of the signal, which is in general called tensor tomography (TT). The most established technique in this category is diffusion magnetic resonance imaging, also called diffusion tensor imaging, which is widely used to study the 3D arrangement and orientation of neurons. In the case of X-rays, TT has been demonstrated for DDF (Malecki *et al.*, 2014 ▸; Kim *et al.*, 2020 ▸), SAXS (Liebi *et al.*, 2015 ▸; Schaff *et al.*, 2015 ▸; Liebi *et al.*, 2018 ▸; Gao *et al.*, 2019 ▸; Nielsen *et al.*, 2023*b* ▸) and WAXS (Grünewald *et al.*, 2020 ▸). Other related tomography approaches which can be considered as TT include probing magnetic field directions with circularly polarized X-rays (Donnelly *et al.*, 2017 ▸) or polarized neutrons (Sales *et al.*, 2017 ▸).

The acquisition and analysis of TT data is a non-trivial undertaking, creating a barrier for the wider adoption of these powerful techniques. In response to this challenge, specifically with regard to the analysis of such data, we here introduce the software package *MUMOTT* for the reconstruction of TT data. While the current implementation supports the cases of SAXS and WAXS, the framework offers the possibility of including other modalities in the future. In the following, we first provide a brief overview of the methodology (Section 2) before describing the structure and functionality of *MUMOTT* (Section 3). Finally, we give a short outlook on potential future additions and developments (Section 4).

## 2. Methodology

SAXS- and WAXS-TT are conceptually similar to XRD-CT. Specifically, in both techniques the sample is raster-scanned through a focused beam to produce a number of 2D projections, varying the sample orientation between each projection. Unlike XRD-CT, SAXS- and WAXS-TT work with azimuthally regrouped detector images where the intensity of the scattered X-rays in a number of azimuthal bins is recorded rather than a single azimuthally integrated intensity. The width of the azimuthal bins depends on the desired angular resolution of the reconstruction. The azimuthal regrouping can be done with a number of freely available software tools such as *pyFAI* (Kieffer *et al.*, 2020 ▸) and *matfraia* (Jensen *et al.*, 2022 ▸). The experimental data thus form a five-dimensional data set consisting of the tomographic rotation, the two directions of the raster scan grid, the scattering angle 2θ and the azimuthal angle φ. *MUMOTT* deals with the transformation of such a five-dimensional data set into a six-dimensional reconstruction, consisting of a three-dimensional voxel map containing a three-dimensional reciprocal-space map (3D-RSM) in each voxel.

We assume that the data have already been corrected for various experimental errors pertaining to solid angle, geometric distortions and polarization. To account for the effect of absorption by the sample, the collected data can be normalized by the transmitted intensity, as is common practice in XRD-CT. Especially at small scattering angles, this makes it possible to carry out reconstructions even with low sample transmission coefficients (∼1% has been demonstrated), assuming sufficient incident flux. The measurement of the transmitted beam intensity can be done using either a semi-transparent beam stop, a diode mounted on the beam stop or a fluorescence measurement (Pauw, 2013 ▸). Alternatively, synthetic transmission data can be calculated via an absorption CT reconstruction (Grünewald *et al.*, 2023 ▸).

**Figure 1:**
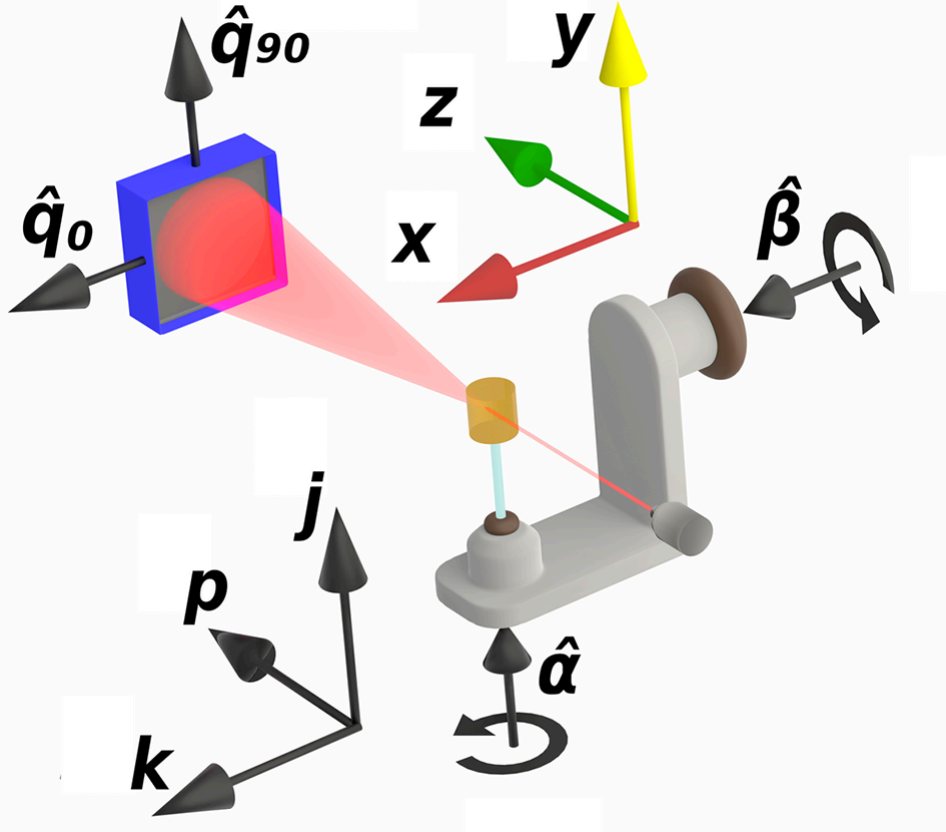
Illustration of vectors defining the experimental geometry in the laboratory coordinates (*i.e.* at α = β = 0).

The experiment is described in a coordinate system defined by the voxel grid of the sample and the three orthogonal basis vectors 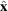, 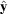 and 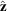. Typically, these vectors are chosen to conform to the convention of the beamline where the experiments were performed, such that the sample-fixed coordinates correspond to the laboratory coordinates when the goniometer angles are zeroed. The geometry of the instrument is defined by specifying a number of unit vectors in these laboratory coordinates. These vectors include the beam direction 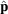 (also called the projection vector), the two orthogonal directions of the raster scan 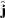 and 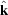, and two vectors describing the origin and the positive direction of the azimuthal integration, 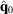 and 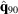, respectively, defined by the equation 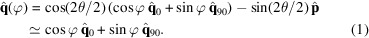
This equation gives the normalized scattering vector 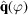 probed by each detector segment as a function of the scattering angle 2θ and the detector azimuth angle φ. The second line gives a useful approximation valid for small scattering angles. The sample can be rotated by a goniometer, and the rotation of the sample goniometer at a given setting labeled *s* results in a rotation matrix **R**_*s*_. Typically, the goniometer is constructed of two orthogonal rotation stages: an inner ‘rotation’ stage and an outer ‘tilt’ stage. The full rotation is then defined by a pair of rotation angles α and β with corresponding rotation axes 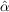 and 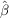, such that 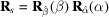. While all these vectors may be chosen freely in *MUMOTT* (under the restriction that certain vectors are orthogonal to certain other vectors), we work in a standard geometry in this paper, given by the choices listed in Table 1 ▸ and visualized in Fig. 1 ▸.

**Figure 2:**
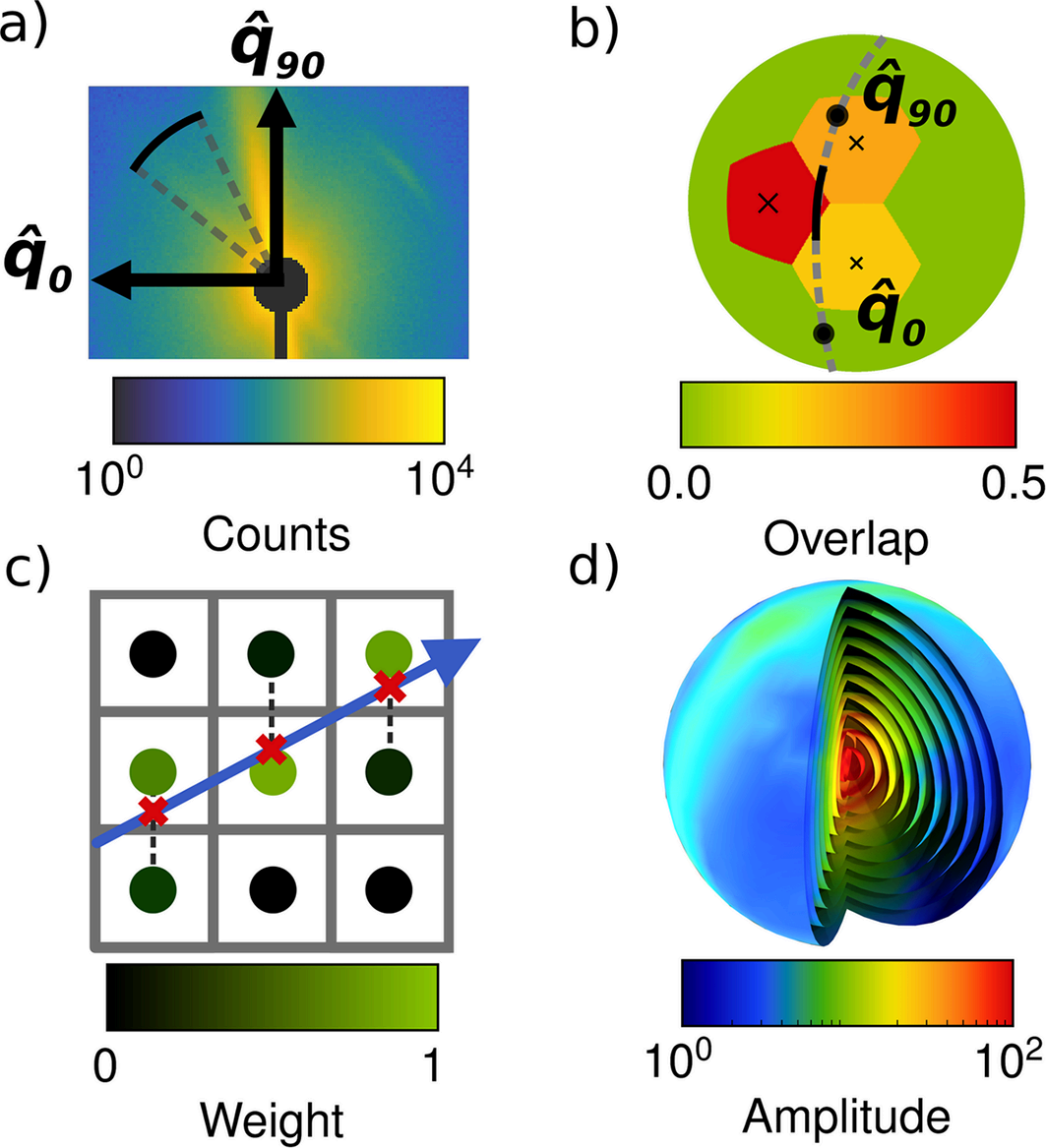
(*a*) Layout of vectors and angles on the detector. A single detector segment is marked with a thick black line. (*b*) Integrated basis function values *B*_*sc*,*i*_ plotted in a stereographic projection. The solid black arc corresponds to the single detector segment marked in panel (*a*). (*c*) Computed probing of each voxel by bilinear interpolation. (*d*) Splitting of a 3D-RSM into a stack of 2D-RSMs at fixed *q* lengths.

The scattering from a given voxel (*x*, *y*, *z*) is proportional to a characteristic function 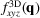 called the 3D-RSM. In the context of SAXS-TT, the RSM is the Fourier transform of the auto-correlation function of the electron density taken over a small volume. For the purpose of reconstruction, we consider one ‘shell’ of reciprocal space at a time, and the 3D-RSM is built up by reconstructing and stacking successive 2D shells [sketched in Fig. 2 ▸(*d*)]. For one such shell we consider the function 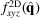, which depends only on the *direction* of the scattering vector. This function is modeled by a sum of basis functions, 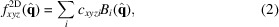
where 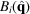 are the basis functions (see Section 3.3 below) and *c*_*xyzi*_ are the unknown expansion coefficients that we want to find.

The function 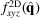 is described in sample-fixed coordinates such that, at a rotation of the sample given by **R**_*s*_, the detector segment at the angle φ measures the component 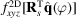, where the superscript T denotes the matrix transpose. In the normal setting, the detector is split into a number of evenly spaced segments indexed by *c*, covering either the full 360° for WAXS or 180° for SAXS. Each detector segment [Fig. 2 ▸(*a*)] is defined by a start angle φ_*c*,start_ and an end angle φ_*c*,end_. As such, the detector segment probes the average of the scattering function within this interval given by the integral 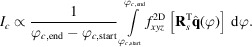
By inserting equation (2) into the above, we define the constants [Figs. 2 ▸(*a*) and 2 ▸(*b*)] 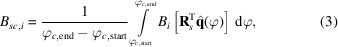
which describe how much each basis function scatters in the direction measured by a given detector segment, illustrated in Fig. 2 ▸(*a*). This is an integral over a single scalar variable, which can be numerically evaluated by standard methods of quadrature.

To complete the forward model, we have to sum the intensity contributions from all voxels in the path of the incident beam. At a given position of the raster scan and rotation of the goniometer, only the voxels that are illuminated by the beam contribute to the measured scattering. A given voxel is indexed by the three integers *x*, *y* and *z*. At a given setting of the sample goniometer the position of the voxel is 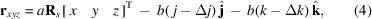
where *j* and *k* are integer indices of the raster scan, *a* is the step size of the cubic voxel grid, *b* is the step size of the 2D raster scan, and Δ*j* and Δ*k* are offsets caused by parasitic movements of the sample stage during rotation. Typically, the resolution of the reconstruction is matched with the raster scan such that *a* = *b*.

Finally, to include the scattering from all probed voxels, we introduce the factor *P*_*sjk*,*xyz*_ which describes how much the *xyz* voxel overlaps with the incoming beam at the position *j*, *k* of the raster scan at the goniometer setting *s*. *P*_*sjk*,*xyz*_ takes a value between 0 and 1, with the value 0 for any voxel that does not intersect the X-ray beam. Using this factor, we can now write the scattered intensity as a sum over all voxels in the voxel grid: 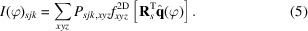


Fig. 2 ▸(*c*) gives a graphical interpretation of the *P*_*sjk*,*xyz*_ coefficients. By combining equations (2) and (5) we can now write up the full forward model for TT: 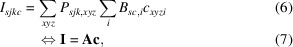
where on the second line we have defined the data vector **I**, the system matrix **A** and the coefficient vector **c** in order to write the problem in linear algebra terms. The system matrix has the block matrix structure 
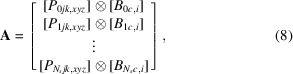
where ⊗ is the Kronecker product. Note that the system matrix does not factorize into a projection part and a reciprocal-space part, as both the projection operator and the basis function matrix depend on the orientation of the sample. This structure highlights the difference between tensor tomography and many other multi-modal tomography techniques such as XRD-CT, X-ray fluorescence tomography (de Jonge & Vogt, 2010 ▸), time-resolved tomography and spectral tomography (Shikhaliev, 2008 ▸), where the real-space projection operation and the mapping of the other modalities are decoupled. This prevents the use of many techniques that rely on this factorization, such as principal component analysis methods (Gao *et al.*, 2021 ▸).

With the forward model defined, we can now formulate the inversion as the solution of a minimization problem: 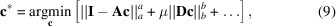
where 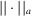 and 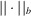 are two, potentially identical, vector norms, μ is a regularization parameter, and **D** is a weight matrix. The ellipsis indicates that more regularization terms of the same form as 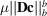 may be added.

## 3. Implementation

*MUMOTT* is written in Python, with performance-critical parts implemented using the *numba* package (Lam *et al.*, 2015 ▸) for CPU and GPU acceleration in order to balance computational efficiency, portability and maintainability. It also depends on *NumPy* (Harris *et al.*, 2020 ▸), *SciPy* (Virtanen *et al.*, 2020 ▸), *scikit-image* (Van der Walt *et al.*, 2014 ▸) and *colorcet* (Kovesi, 2015 ▸). The package is extensively documented and the documentation is available online at https://mumott.org and at https://doi.org/10.5281/zenodo.7919448, including various examples in the form of *Jupyter* notebooks.

A variety of common tasks pertaining to data alignment and reconstruction are accessible via functions that provide a rather simple yet customizable interface. These functions represent ‘pipelines’ (Section 3.4) and are intended to serve as the primary interface for most users.

The pipeline functions combine a number of individual tasks and components, which are represented via objects and are part of the underlying object-oriented framework (Section 3.5). Through the latter, advanced users and developers can customize, adapt and extend the functionality of *MUMOTT. MUMOTT* is released under the Mozilla Public License Version 2.0 and developed as free and open source software, inviting the contributions of other groups and developers.

In the following, we first provide a short demonstration of the workflow (Section 3.1) before addressing basis sets (Section 3.3), several common pipelines (Section 3.4), the underlying object-oriented framework (Section 3.5) and computational efficiency (Section 3.6).

### 3.1. Workflow

**Figure 3:**
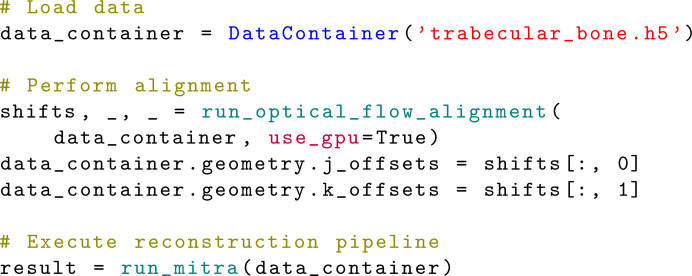
Minimal example of a reconstruction workflow using the MITRA pipeline (Section 3.4.1).

Figs. 3 ▸ and 4 ▸ show examples of simple workflows in *MUMOTT* for reconstructing a voxel map of 2D-RSMs from experimental data. In the following sections, we explain each of the steps in this process.

#### 3.1.1. Loading the data

The acquired data (after beamline-specific preprocessing) are handled using a DataContainer, which is created by loading an HDF5 file (Table 2 ▸) that contains the azimuthally regrouped data for one *q* bin of the experiment. While a full experimental data set containing a detector frame for each scan position can be quite large, commonly of the order of hundreds of gigabytes, a single *q* bin of the azimuthally regrouped data is usually hundreds of megabytes to a few gigabytes. The prepared data files can contain the geometry data and sample offset information or only the data.

#### 3.1.2. Definition of the geometry

The geometry is defined by the vectors listed in Table 1 ▸, which can be given in any consistent coordinate system. In the examples shown, the full geometry data are already contained in the data file (and hence the DataContainer), but in general it is possible to override certain parameters after loading.

#### 3.1.3. Aligning the data

Before a meaningful reconstruction can be carried out the data must be aligned, which means calculating the offsets defined in equation (4). To this end, *MUMOTT* provides several pipelines that use the transmission measurement or the average scattering to correct misalignment between each projection that occurs due to parasitic movements during acquisition. In the examples shown here, we use the function that implements the optical flow alignment procedure (Odstrčil *et al.*, 2019 ▸), which relies on center-of-mass and tomographic consistency techniques. The alignment functions return, most importantly, the shifts that are needed for aligning the data. These values are then used to override the offsets stored in the DataContainer object.

#### 3.1.4. Defining the reconstruction model

**Figure 4:**
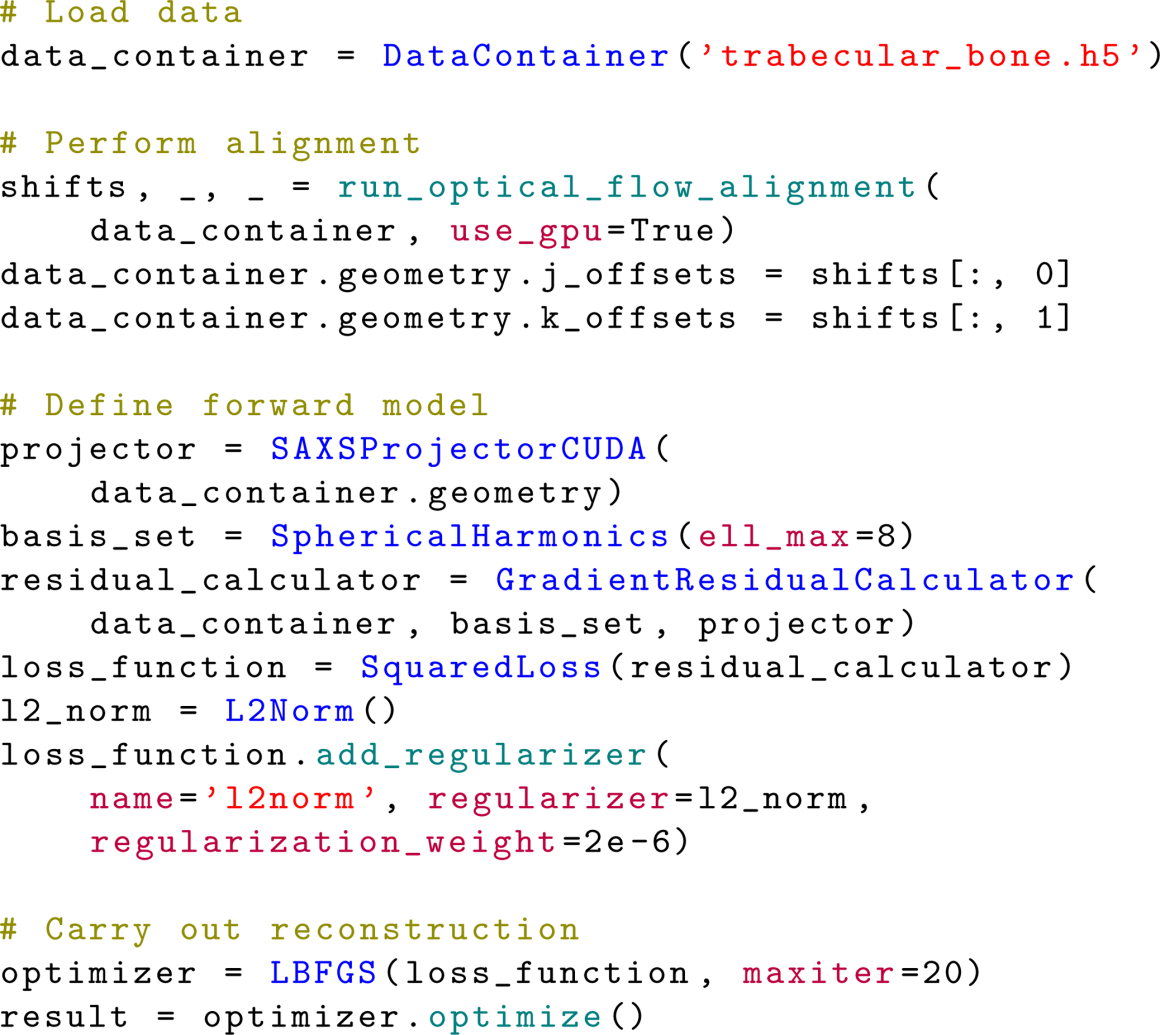
Extended example of a reconstruction workflow using a Tikhonov (*L*_2_) regularized least-squares model and spherical harmonics as basis functions with the object-oriented interface (Section 3.5).

The reconstruction model is defined by the choice of basis functions, the form of the cost function and the regularization terms. A large number of different algorithms can be constructed by combining these three choices. The simplest approach is to utilize one of the existing pipelines (Section 3.4), as illustrated by the first example (Fig. 3 ▸) in which the modular iterative tomographic reconstruction algorithm (MITRA) pipeline (Section 3.4.1) is used. Alternatively, one can configure a reconstruction model using the individual objects that represent the different components. This approach is demonstrated by the second example (Fig. 4 ▸), where we choose a basis of spherical harmonics in combination with a squared-difference loss function and Tikhonov (*L*_2_) regularization.

#### 3.1.5. Minimizing the loss function

Once the loss function is defined, the optimization problem can be solved using one of a number of optimization routines. While this step is included in the case of the predefined reconstruction pipeline in the first example (Fig. 3 ▸), it needs to be explicitly specified when constructing the workflow as in the second example (Fig. 4 ▸), where we use the gradient-based LBFGS (Liu & Nocedal, 1989 ▸) optimizer. In the case of regularized models, one should then perform a sweep of the regularization parameter space in order to determine (a) sensible regularization parameter(s).

### 3.2. Deriving standard quantities from the output

The result of a tensor tomography reconstruction is an array of optimized coefficients 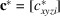 which are the voxel-by-voxel expansion coefficients of the local 2D-RSM shells in terms of the specific basis functions. In general, the coefficients can be interpreted using the corresponding basis set to compute latitude–longitude maps of the 2D-RSM shells, and reconstructions of several *q* bins can be combined to construct 3D-RSMs from these maps. A number of derived quantities are conventionally used for evaluation and visual­ization of reconstructions, and these can be calculated efficiently from the coefficients without needing to compute latitude–longitude maps. Note that we define here the derived quantities which are part of the output structure of *MUMOTT*. Additional quantities can be calculated from the array of optimized coefficients, depending on the basis function.

The mean scattering intensity, also called the isotropic intensity, is defined as 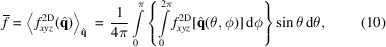
where θ and ϕ are a pair of polar coordinates for the unit sphere.

A 2D-RSM can also be expanded in tensor components. The rank-2 tensor is of particular interest because it allows easy computing of primary directions given by the eigen­vectors of the matrix. The second-moment tensor is a 3 × 3 matrix with elements 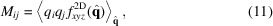
where *q*_*i*_ are the *x*, *y* and *z* components of 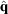 for *i* = 1, 2 and 3, respectively.

**Figure 5:**
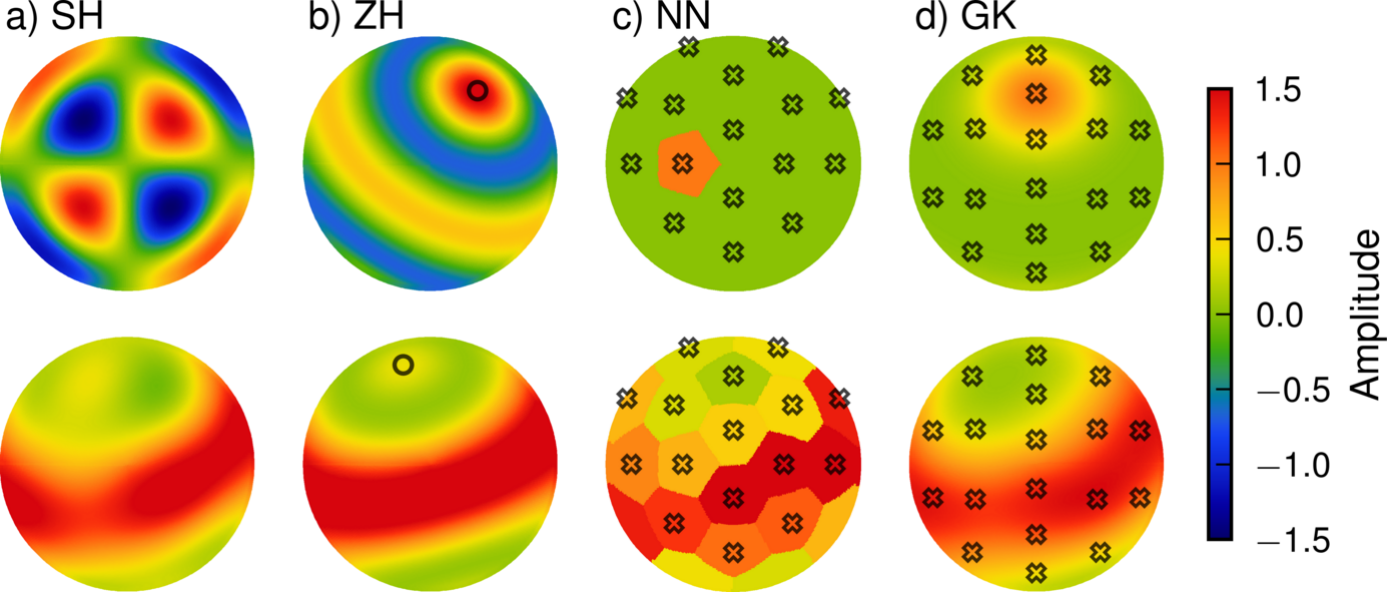
Comparison of (*a*) SH, (*b*) ZH, (*c*) NN and (*d*) GK basis sets. The upper row shows a single basis function for each basis set. The lower row shows the RSM at a single *q* of a single voxel of a reconstruction using each of the four basis sets. The crosses in panels (*c*) and (*d*) show the grids which are part of the definition of the NN and GK basis sets. The directions used to define the NN model are the face centers of the truncated icosahedron. The directions used in the GK model are given by a modified Kurihara mesh. The circles in panel (*b*) indicate the symmetry axes.

For many samples, a main orientation, such as a fiber symmetry axis of the nanostructure, can be defined for each voxel. The rank-2 tensor provides a means of efficiently computing this direction through its eigendecomposition. However, the interpretation of the main orientation of the nanostructure depends on its scattering characteristics. In structures where a single direction of strong scattering is expected at two opposite poles, the main orientation is the eigenvector corresponding to the largest eigenvalue. Similarly, for samples where a ring equatorial band with strong scattering is observed (*e.g.* the structure displayed in Fig. 5) the eigenvector corresponding to the smallest eigenvalue should be chosen. This provides a fast and noise-tolerant approach to finding the main nanostructure orientation, except in cases where the rank-2 term vanishes. The latter can occur, *e.g.*, in Bragg scattering from cubic symmetric materials, where more advanced approaches are needed.

Another quantity frequently used to describe the anisotropy (Basser & Pierpaoli, 1996 ▸) of tensor tomography is the fractional anisotropy (FA), which can be computed from the eigenvalues of the 2nd moment tensor:
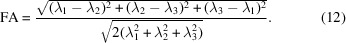
Here, λ_1_, λ_2_ and λ_3_ are the three eigenvalues of the second moment tensor. FA = 0 for perfectly isotropic scattering, and it reaches a maximum value of 1 when there is strong scattering in one direction and the scattering goes to zero in the orthogonal directions. Other values can be calculated from the coefficients to describe the anisotropy, also referred to as the degree of orientation, and these are described elsewhere (Liebi *et al.*, 2015 ▸; Nielsen *et al.*, 2023*b* ▸; Nielsen *et al.*, 2024 ▸).

### 3.3. Basis sets

The most notable difference between different reconstruction algorithms is the choice of basis functions. Fig. 5 ▸ shows a comparison of the optimized 2D-RSM shell of a single voxel of the same sample using four different basis-set types.

#### 3.3.1. Spherical harmonics

The spherical harmonics (SH) are a set of orthogonal polynomials that derive from the solution to the Laplace equation in spherical coordinates. Any function on the unit sphere can be represented by an infinite expansion in spherical harmonics, but in practice the expansion must be truncated at some finite order. Such a finite expansion in spherical harmonics is called a band-limited spherical function and can be used to represent the 2D-RSM (Nielsen *et al.*, 2023*b* ▸). The SH basis set is fully defined by the band limit ℓ_max_ at which the expansion in spherical harmonics is truncated. This sets the resolution of the narrowest diffraction features that can be reconstructed to approximately 2π/ℓ_max_ radians.

#### 3.3.2. Nearest neighbors

A nearest-neighbors (NN) basis set uses a set of NN indicator functions. This model is therefore defined by a grid of orientations alone and the resolution is set by the distance between grid points. This basis set can be used to emulate the algorithm first presented by Schaff *et al.* (2015 ▸), which splits the TT problem into a set of independent scalar tomography problems.

#### 3.3.3. Gaussian kernels

Like the NN basis set, the Gaussian kernels (GK) basis set is defined by a grid of orientations, but instead of indicator functions it uses smooth spherical Gaussian functions, rotated to be centered on the various grid orientations. It therefore needs one extra parameter to define the basis set, namely the width of the kernel. The GK basis set has many of the same properties as the NN basis set but unlike the former results in smooth RSMs. The resolution depends on both the distance between grid points and the kernel width. Such spherical kernels are commonly used in texture analysis, where the specific function used is referred to as the Bunge normal distribution (Bunge, 1969 ▸) to distinguish it from several other Gaussian-shaped kernel functions that are frequently used.

#### 3.3.4. Zonal harmonics

The axially symmetric method established by Liebi *et al.* (2015 ▸) uses a zonal harmonics (ZH) basis set and thereby differs from the other methods implemented in *MUMOTT* by having a nonlinear forward model. This requires a separate workflow involving a specialized calculator for the gradients and optimizer. To enable high-order expansions, simplify the code, and ensure interoperability between the ZH and SH workflows, rotations and gradients are calculated in coefficient space using Wigner *D*-matrices. This allows orders up to ℓ_max_ = 100 in the current implementation, although orders higher than ℓ_max_ ≃ 30 are difficult to handle in practice due to the large number of coefficients. Details of the implementation are given by Carlsen *et al.* (2024 ▸).

The nonlinearity of the forward model in the ZH approach makes the loss function non-convex, which renders the optimization problem more challenging. Approaches to overcoming this difficulty include regularization of the angle parameters and smoothing of the gradient (Liebi *et al.*, 2018 ▸), as well as the use of an ensemble of randomized starting points (Nielsen *et al.*, 2023*b* ▸). In *MUMOTT* we use a starting guess provided by a different reconstruction algorithm to determine the symmetry direction.

### 3.4. Pipelines

*MUMOTT* provides various pipelines that implement reconstruction and alignment workflows. The former include both ‘standard’ and asynchronous pipelines. The standard pipelines can be run using both CPU and GPU resources and are usually highly customizable. The asynchronous pipelines are optimized for GPU resources and thus speed, and are usually slightly less adjustable. They employ asynchronous execution on the GPU to avoid the overhead caused by transferring data between the CPU and GPU.

#### 3.4.1. Standard reconstruction pipelines

The simultaneous iterative reconstruction technique (SIRT) is a popular reconstruction algorithm thanks to its inherent regularizing properties that result from semi-convergence (Elfving *et al.*, 2012 ▸) and the small number of tunable parameters. It has previously been used for tensor tomography by *e.g.* Schaff *et al.* (2015 ▸) and Kim *et al.* (2020 ▸). In *MUMOTT* a traditional approach to SIRT is implemented in the SIRT pipeline.

While the SIRT algorithm is not conventionally stated as a minimization problem, it has been shown that it is equivalent to a specific preconditioned gradient-descent weighted least-squares optimization (Gregor & Fessler, 2015 ▸). Through this re-formulation, the basic SIRT reconstruction becomes com­patible with various regularizers. The weight-preconditioner approach employed in this form of SIRT can also be extended to the RSM given by equation (3). This approach is implemented in the MITRA pipeline, which permits arbitrary regularizers and basis sets to be used, as well as Nesterov momentum acceleration.

The spherical integral geometric tensor tomography (SIGTT) pipeline sets up the basic reconstruction model using an SH basis set, a squared-difference loss function and regularization via a finite-difference Laplacian filter (Nielsen *et al.*, 2023*b* ▸). The optimization problem is solved with the LBFGS-B algorithm and uses a stop criterion based on the relative change in the loss function.

The discrete directions (DD) pipeline emulates the reconstruction technique used by Schaff *et al.* (2015 ▸), which splits the tensor reconstruction into a set of independent scalar reconstructions using the NN basis set. The pipeline employs the SIRT algorithm for the individual scalar reconstructions. DD has the practical advantage of needing less VRAM than methods which reconstruct the entire RSM at once, as it only loads one scalar component onto the GPU at a time.

#### 3.4.2. Asynchronous pipelines

These pipelines, optimized for GPU execution and speed, include a tensor SIRT pipeline, which is similar to MITRA without Nesterov momentum. In addition, there is momentum total variation reconstruction (MOTR), which is essentially the default MITRA pipeline with *L*_1_ and two-sided total variation regularization. Finally, robust and denoised tensor tomography (RADTT) optimizes for the Huber norm with two-sided total variation regularization through Nesterov accelerated gradient descent. This last pipeline requires fine-tuning of the configuration in order to converge reasonably well, but once an appropriate step size and smoothing terms are found, it is relatively robust against noise.

There are also sparse versions of the asynchronous pipelines, which use a modified version of the John transform that calculates the reciprocal-space and real-space projection operations simultaneously within one kernel, using a sparse approximation to the reciprocal-space mapping. This is not necessarily faster than computing the two mappings separately (unless the representation is very sparse, such as only mapping one basis function to each segment). It does, however, use less VRAM, as it is not necessary to store the intermediate result between carrying out the John transform and carrying out the reciprocal-space mapping.

#### 3.4.3. Alignment

The objective of alignment is to determine the offsets Δ*j* and Δ*k* of equation (4) that result from parasitic movement and misalignment of the goniometer and drift during the experiment (Frank *et al.*, 1992 ▸). The alignment step is essential in tomography, as any misalignment will be reflected as an artifact in the tomographic reconstruction. There are many algorithms to solve this problem, leading to sub-pixel alignment accuracy, taking into account various experimental systems and data.

**Figure 6:**
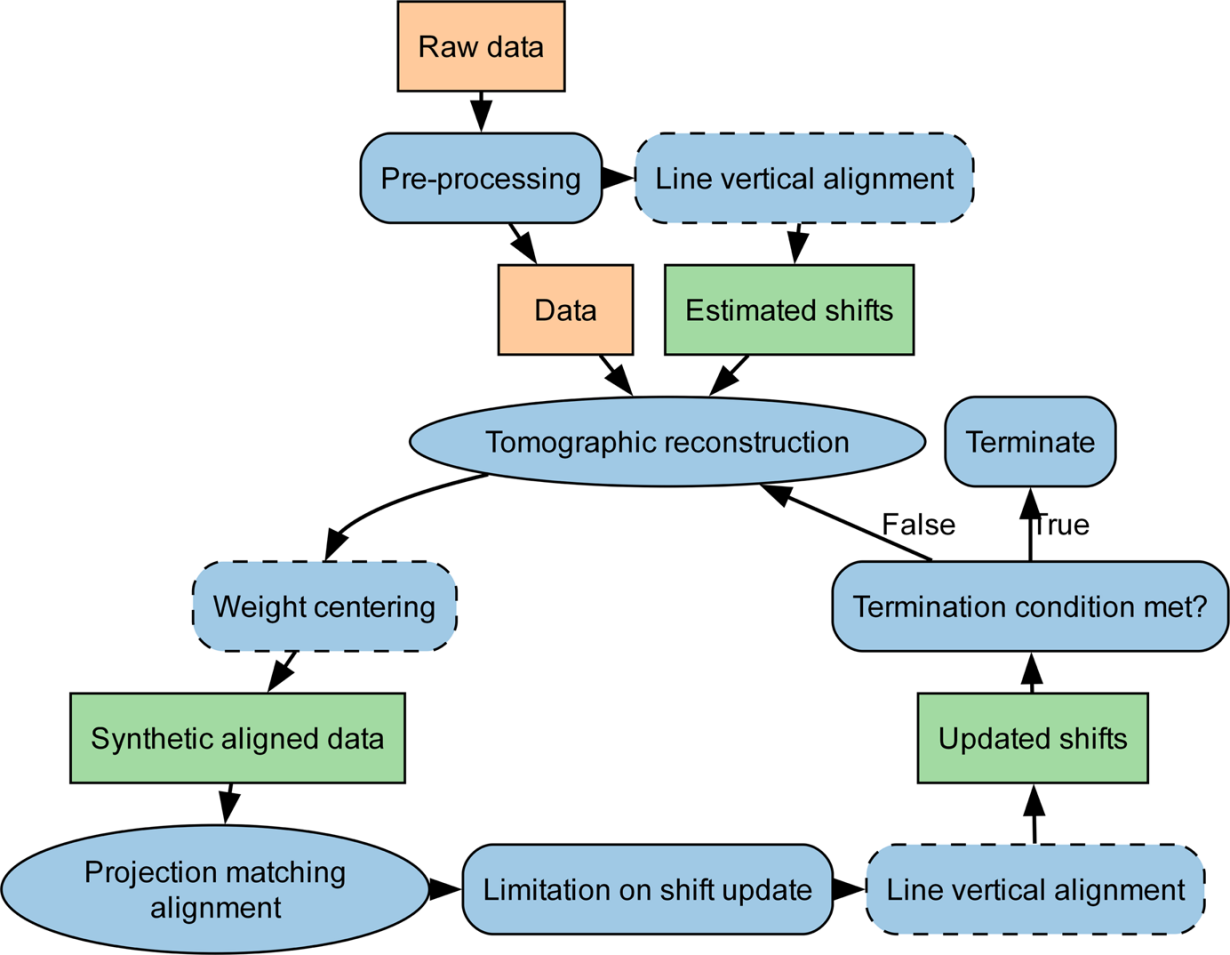
Alignment pipeline workflow. Steps shown with dashed outlines apply only for the optical flow alignment pipeline.

*MUMOTT* currently provides two alignment pipelines. Both algorithms typically work with the transmitted intensity data stored in the DataContainer or another isotropic signal such as the azimuthally integrated intensity. They iteratively update the offsets for each projection by reconstructing the absorption tomogram via a projector. The overall workflow is shown in Fig. 6 ▸.

The *phase matching alignment* pipeline is based on cross-correlation and follows Guizar-Sicairos *et al.* (2008 ▸). Cross-correlation alignment has been proven for continuous objects in electron microscopy tomography by Guckenberger (1982 ▸) and has been widely used since. The principle is to determine the offsets by means of correlation functions formed from image pairs of the projections, comparing the centers of mass of the image pair correlation peaks. This method is fast and can provide sub-pixel accuracy for data with small misalignment.

When the data exhibit misalignment of multiple pixels, the cross-correlation alignment alone can struggle to find the appropriate coordinate transformation. For such cases, *MUMOTT* provides the *optical flow alignment* pipeline, which implements a toolbox algorithm based on the work of Odstrčil *et al.* (2019 ▸). This approach uses multiple successive and interconnected alignment procedures, including optical flow projection, matching alignment, line vertical alignment and weight centering. The method is tunable through various parameters and filters and is therefore able to align extremely misaligned data. It thereby provides an approach that is usable for a larger variety of experimental data.

**Figure 7:**
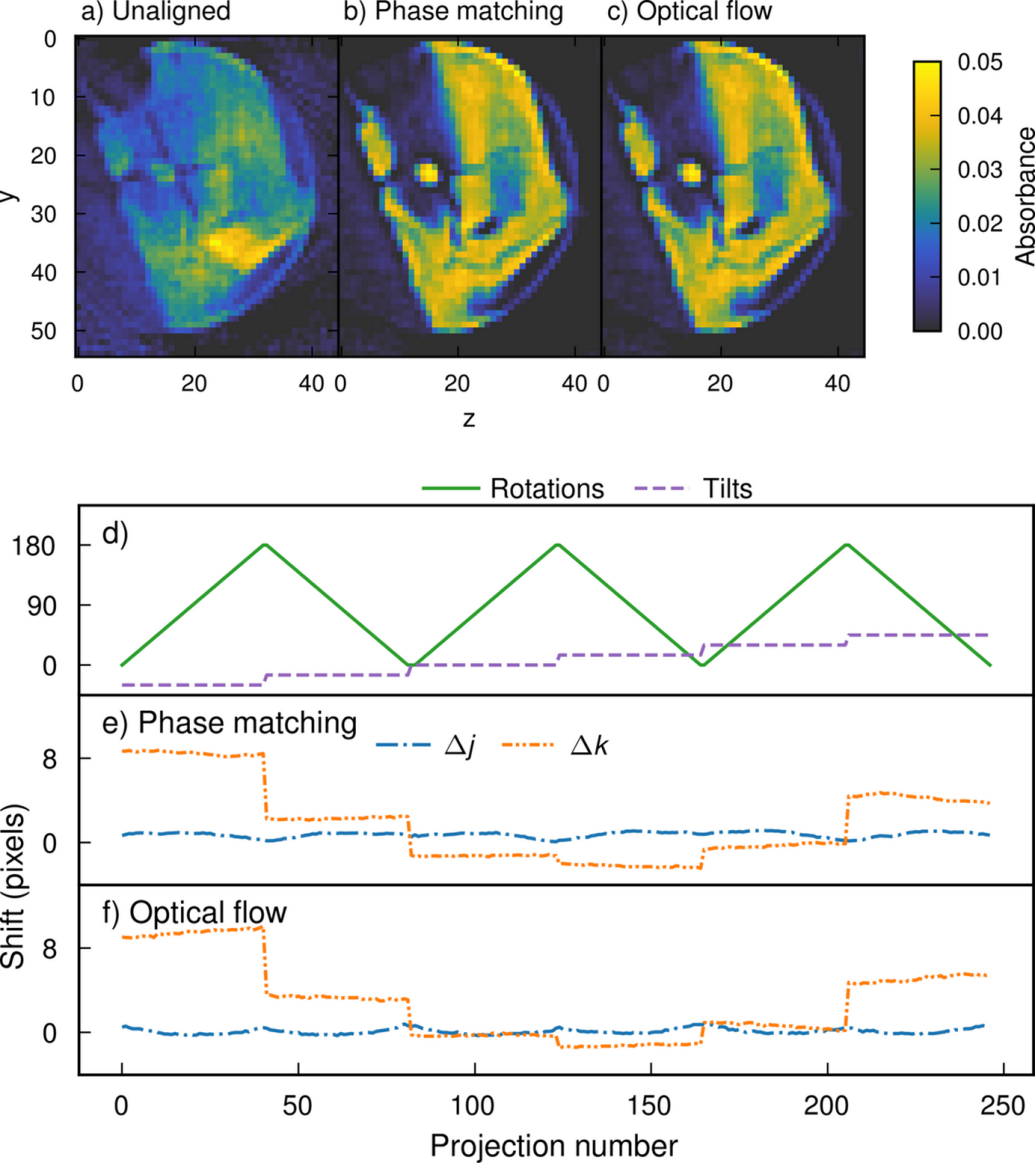
Slices from absorption reconstructions, (*a*) before alignment, and (*b*) and (*c*) after alignment with (*b*) the phase matching method and (*c*) the optical flow method. The projections have been sorted so that the projection directions of neighboring projections are as close to each other as possible. (*d*) Rotations and tilts. (*e*) and (*f*) Alignment offsets from (*e*) the phase matching method and (*f*) the optical flow method. Note how the rotations correlate with changes in Δ*j*, whereas the tilts correlate with changes in Δ*k*. In this case the offsets result from misalignment of the goniometer’s rotation axes with the sample center.

Examples of alignment results with the two pipelines are shown in the case of a publicly available experimental data set from trabecular bone in Fig. 7 ▸ (Nielsen *et al.*, 2023*a* ▸).

### 3.5. Object-oriented framework

**Figure 8:**
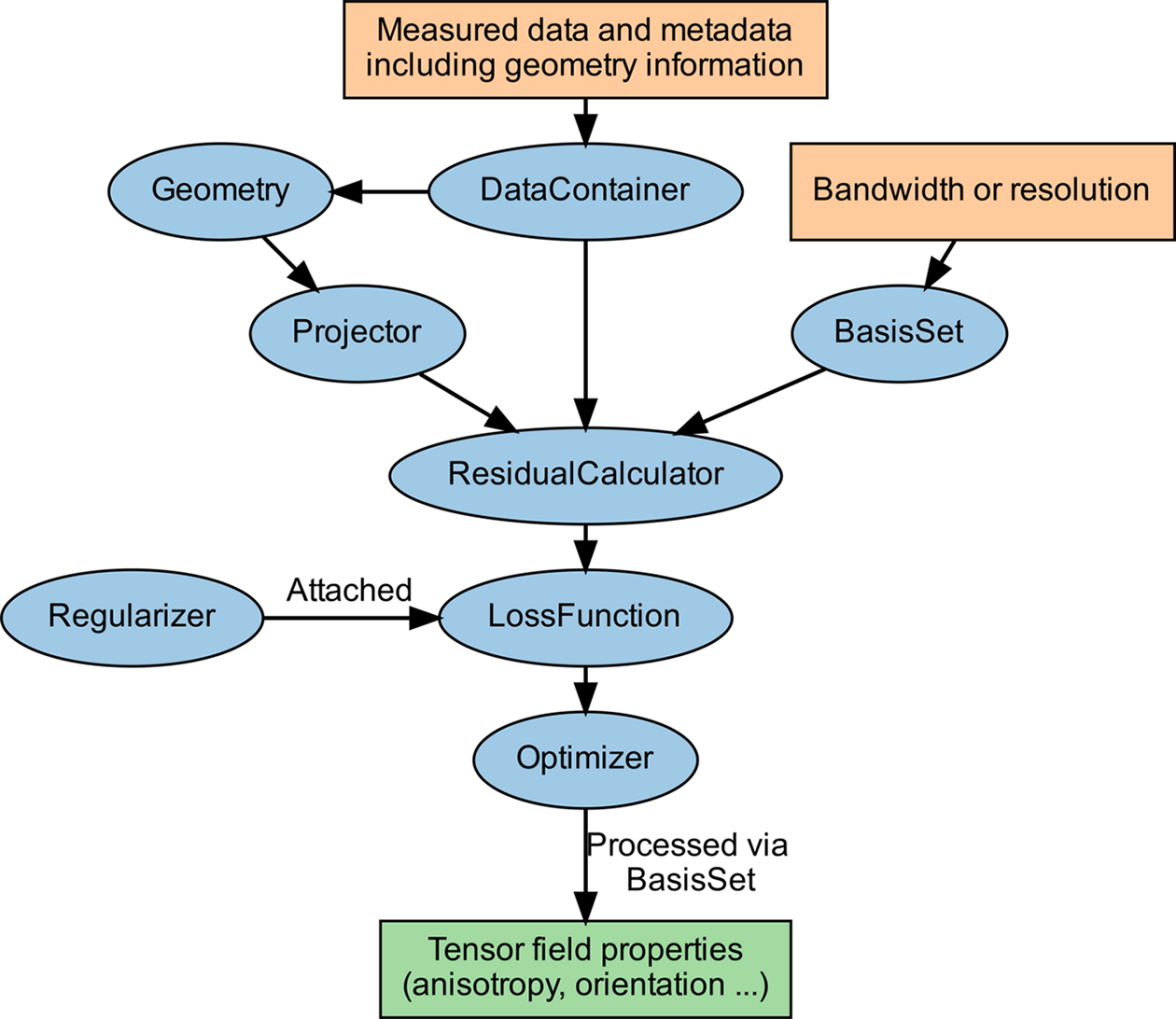
Outline of the object-oriented framework in *MUMOTT*. Orange boxes show input parameters and data provided by the user, blue ovals show objects, the green box shows the output, and arrows indicate instances of objects interacting with one another.

The internal architecture of *MUMOTT* consists of an object-oriented framework with some elements of functional programming. The structure of the framework is described in Fig. 8 ▸. Many objects are safely mutable after instantiation and employ hashes of their mutable properties to track the state of linked instances. This is used internally to trigger recomputing of derived properties when required.

#### 3.5.1. Data and geometry

The DataContainer is the owner of the input data, which are stored in HDF5 format. The input (measurements and geometry metadata) is stored as a list of projections, indexed by the direction index *s* as given in equation (5), and the measured tensor tomographic data can be accessed as a four-dimensional array indexed by [*s*, *j*, *k*, *i*] as in equation (7). The information related to geometry is stored in a Geometry object, which is directly linked to the list of projections attached to the DataContainer. Thus, if a projection is removed from the list, this will be reflected in the corresponding geometry data being removed from the Geometry instance. The Geometry object stores the basis vectors of the system, *i.e.* (**p**, **j**, **k**, **q**_0_, **q**_90_, 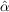, 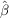) listed in Table 1 ▸ and shown in Fig. 1 ▸. The vectors must be specified in the laboratory coordinate system, which coincides with the sample-fixed coordinates (*x*, *y*, *z*) when **R**(*s*) = **I**, *i.e.* the identity transform. A rotation operator is then specified, **R**(*s*), which may be given as a rotation matrix or as an axis–angle quadruplet 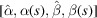. Using the rotation operator, vectors in the sample-fixed coordinates are dynamically computed for each *s*. This information can be specified in the input data file or by the user through direct modification of the Geometry object (see *e.g.* Fig. 3 ▸).

#### 3.5.2. Projectors and basis sets

The Projector and BasisSet classes contain the methods and properties needed to compute the forward model defined in equation (7) and its adjoint. The Projector objects depend on a Geometry object and employ routines implemented using the *numba* package (Lam *et al.*, 2015 ▸) to compute the spatial part of the transform, *i.e.* the matrix elements *P*_*sjk*,*xyz*_ in equation (5). This is implemented for both CPU- and GPU-based computation, the latter using the *numba* interface for CUDA. The implementation employs an approach based on Joseph’s method (Joseph, 1982 ▸) using bilinear interpolation of the field for the forward model and the projection for the adjoint computation, respectively, following the work of Xu *et al.* (2010 ▸) and Palenstijn *et al.* (2011 ▸).

The BasisSet evaluates the constants *B*_*sc*,*i*_ in equation (3) for the respective basis **B** (Section 3.3) using the provided detector geometry and rotation operator 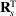. The integral is evaluated using adaptive Newton–Cotes quadrature or approximated using the central angle of each segment. In addition, the BasisSet provides a routine for computing various properties of reconstructed tensors, such as the orientation as defined by the rank-2 tensor component of the field, the spherical mean, the variance and the relative anisotropy (the spherical standard deviation normalized by the mean).

#### 3.5.3. Residual calculation

The ResidualCalculator is a managing object which takes a DataContainer, Projector and BasisSet and uses them to compute residuals. It tracks the current reconstruction, *i.e.c*_*xyzi*_. In other words, for the data *D*_*sjkc*_ and the current reconstruction *c*_*xyzi*_ it computes 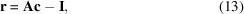
where **r** and **I** are flattened vectors of the residual and data matrices, respectively, using the notation introduced in equation (7). It also computes the gradient of a residual norm, which is used by the gradient-based optimization algorithms implemented in *MUMOTT*.

A special ZonalHarmonicsGradientCalculator is defined to be used as a part of the ZH workflow. It is used to map a list of ZH coefficients and two angle coordinates onto the space of all spherical harmonics (up to a maximum order) in the sample coordinate system and to compute gradients with respect to the ZH coefficients and the angles.

#### 3.5.4. Optimization

The goal of the optimization is to minimize a LossFunction (also known as an objective function) by tuning the coefficients of the underlying model using an Optimizer. The LossFunction combines a Residual­Calculator with one or several Regularizer instances and can be given a preconditioner to weight the gradient.

There are currently two types of loss function, which support standard least-squares regression (SquaredLoss) and robust regression via the Huber regressor (Huber, 1964 ▸) (HuberLoss).

There are also various regularization options. One can *e.g.* smooth the solution by minimizing the squared *L*_2_ norm of the finite-difference Laplacian operator of the tensor field (Laplacian). It is also possible to smooth the solution in a more robust manner by minimizing the Huber norm of the spatial gradient for each basis-set mode (Total­Variation). While it can be more difficult to obtain convergence with more robust terms, *MUMOTT* can also be configured to use the Huber approximation for small values to improve convergence.

Other Regularizer classes are available to minimize the *L*_2_ and *L*_1_ norms of the tensor field. While the *L*_2_ norm (L2Norm) penalizes large values, which promotes rapid convergence, the *L*_1_ norm (L1Norm) encourages sparse solutions and tends to reduce noise in the solution. Finally, one can also use the Huber norm of the tensor field (HuberNorm), which acts as an L1Norm for large values and an L2Norm for small values, converging more easily than L1Norm. When applied with the SH basis set, the L1Norm and HuberNorm are not rotational invariants and can bias the solution towards certain directions.

In terms of optimizers, *MUMOTT* provides gradient descent with a fixed step size (GradientDescent), with an option to use Nesterov accelerated momentum, as well as the LBFGS-B algorithm for quasi-Newton solution of the optimization (LBFGS). For the ZH workflow (Section 3.3.4) there is both a specialized optimizer (ZonalHarmonics­Optimizer) and a gradient calculator (Zonal­Harmonics­Gradient­Calculator). The former is a basic gradient descent optimizer with a special heuristic rule to determine a safe step size for the angle parameters. Because of the non-convexity of the cost function, it requires a good starting guess for the angles in order to converge to a solution.

### 3.6. Computational efficiency and resource requirements

The computational resources required to perform reconstructions in *MUMOTT* are modest compared with previous implementations due to efficient implementations of the John transform and the use of memory-efficient solvers. The place where a user is most likely to run into problems is the memory requirement for the data set and solution vector, in addition to a few extra similarly sized arrays needed by the solvers. The memory requirement is around a few gigabytes in the most common use cases, but increases with both the size of the voxel grid and the directional resolution. In order to use the GPU implementation, one requires a CUDA-compatible GPU with sufficient VRAM to store an array the size of the solution vector.

In general, the reconstruction is much less resource hungry than the preceding data-reduction steps. However, when conducting sweeps of regularization parameters and full *q*-resolved 3D-RSM reconstructions, the reduced runtime from GPU acceleration has a considerable impact.

Table 3 ▸ compares the run times for different pipelines, platforms and configurations. Each configuration was run ten times on each platform, and the result was obtained by averaging the run times after discarding the first run, to enable on-disk caching to take place. All runs were carried out with a maximum of 20 iterations, although SIGTT converged in 14 or fewer iterations in all cases.

The runs were carried out in separate sequentially run processes, which means that just-in-time compiled kernels were not reused beyond what is automatically cached on disk. This has the largest effect on DD, which creates sub-geometries for each basis function and therefore needs to recompile code to carry out the John transform for each sub-iteration. This adds approximately one second of overhead per basis function. Therefore, DD can perform substantially better than what is apparent from this table when the same geometry is run multiple times in a single process, as may be done for *q*-resolved reconstruction. The time required to load data was not included in the timing to eliminate the dependency on the file systems used for benchmarking.

## 4. Outlook

Various additions and improvements to *MUMOTT* are foreseen for the future. One of the main difficulties of performing tensor tomography experiments at present is the interfacing of the existing data analysis pipelines at the various synchrotron end stations with the reconstruction pipeline. At present, such an integration relies on two intermediate steps. In the first step, the detector images are azimuthally regrouped, which results in a number of new data files containing the azimuthally regrouped intensities that are organized projection by projection, mirroring the order in which the experiment was performed. In the second step, the experimental data set is sliced into *MUMOTT*-compatible HDF5 files as described in Table 2 ▸, which contain the data organized by *q* bins. These extra analysis steps are often slow as they require many read and write operations. On-the-fly reconstructions would require live azimuthal regrouping of detector images and a more efficient data pipeline that allows fast slicing in the *q* dimension.

At present, *MUMOTT* is able to compute various properties of reconstructions and save the results to HDF5 files. The user then has the responsibility for analysis and visualization of the reconstructed quantities. It will be useful to add the option to write to formats compatible with common visualization software packages.

Nielsen *et al.* (2023*b* ▸) used simulated data for the purpose of validation and comparison of various reconstruction methods. Being easily able to generate simulated data in *MUMOTT* would be useful not just for validation but also to plan experiments and to generate synthetic data for training machine learning models.

The splitting of the tensor tomography reconstruction into discrete 2D-RSM shells is a useful simplification that reduces the size of individual reconstruction problems. It would, however, often be advantageous to combine several *q* bins into a single reconstruction to enforce certain types of prior knowledge of the nanostructure on the reconstruction for sample systems where an appropriate model is available. This applies *e.g.* in the case of texture tomography (Frewein *et al.*, 2024 ▸) with Bragg scattering from nanocrystalline materials, where the rotational symmetries of the crystal lattice can be imposed on the reconstruction by performing a combined reconstruction of several *q* bins at once. Also, in the case of fiber scattering, different *q* ranges can contain scattering from different sample orientations, and a combined approach to reconstruction is expected to be able to alleviate missing wedge artifacts in the reconstructions.

## Supplementary Material

Zenodo record for software releases: https://doi.org/10.5281/zenodo.7919448

## Figures and Tables

**Table 1:** Unit vectors defining the experimental geometry and their values in the standard geometry used in previous publications such as Liebi *et al.* (2018 ▸)

Symbol	Standard	Field name
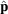	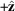	p_direction_0
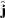	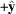	j_direction_0
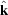	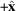	k_direction_0
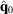	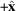	detector_direction_origin
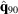	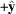	detector_direction_positive_90
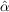	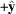	inner_axis
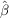	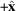	outer_axis

**Table 2:** Outline of the HDF5 file format used by *MUMOTT* The indents indicate the hierarchy of entries; 0 is an entry in the group projections, whereas data is an entry in the group 0 and so on.

Path			Type
p_direction_0			float(3)
j_direction_0			float(3)
k_direction_0			float(3)
detector_direction_origin			float(3)
detector_direction_positive_90			float(3)
inner_axis			float(3)
outer_axis			float(3)
volume_shape			int(3)
detector_angles			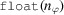
projections			Group
0			Group
data			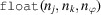
diode			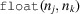
inner_angle			float(1)
j_offset			float(1)
k_offset			float(1)
outer_angle			float(1)
weights			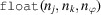
1			Group
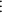			

**Table 3:** Comparison of reconstruction times in seconds averaged across ten runs each for a typical single-*q* data set consisting of 247 projections, each with 65 × 55 pixels and eight detector segments, using different reconstruction pipelines and running on different computers *N* is the number of basis functions per voxel. In all cases, relative uncertainties were smaller than 5% and are omitted to maintain ease of reading. The workstation (WS) data were obtained using an AMD Ryzen 7 3700X processor with eight physical cores, 64 GB of DDR4, 2666 MHz RAM and, for the GPU-accelerated calculations, an Nvidia GeForce RTX 3060 GPU with 12 GB of VRAM. The high-performance computing (HPC) CPU timings were generated using eight top-level threads on a 64-core Intel Xeon Platinum 8358 @ 2.0 GHz CPU with some operations utilizing lower-level multithreading. The HPC GPU timings were obtained on an Nvidia A100 GPU with 40 GB of VRAM and eight threads on 16 cores of a 64-core Intel Xeon Platinum 8358 @ 2.0 GHz CPU.

		CPU		GPU	
Pipeline	*N*	WS	HPC	WS	HPC
SIGTT	6	23	18	9	9
	20	45	29	18	14
	72	108	69	60	36
					
MITRA	18	41	22	13	8
	50	93	45	40	14
	162	271	156	115	37
					
DD	18	81	72	40	43
	50	156	157	101	111
	162	334	392	290	346
					
MOTR	18			9	8
	50			12	10
	162			46	22
